# Enabling the electrocatalytic fixation of N_2_ to NH_3_ by C-doped TiO_2_ nanoparticles under ambient conditions†

**DOI:** 10.1039/c8na00300a

**Published:** 2018-11-21

**Authors:** Kun Jia, Yuan Wang, Qi Pan, Benhe Zhong, Yonglan Luo, Guanwei Cui, Xiaodong Guo, Xuping Sun

**Affiliations:** School of Chemical Engineering, Sichuan University Chengdu 610065 China xiaodong2009@scu.edu.cn; Institute of Fundamental and Frontier Sciences, University of Electronic Science and Technology of China Chengdu 610054 China xpsun@uestc.edu.cn; College of Chemistry, Chemical Engineering and Materials Science, Shandong Normal University Jinan 250014 Shandong China

## Abstract

The conventional Haber–Bosch process for industrial NH_3_ production from N_2_ and H_2_ is highly energy-intensive with a large amount of CO_2_ emissions and finding a more suitable method for NH_3_ synthesis under mild conditions is a very attractive topic. The electrocatalytic N_2_ reduction reaction (NRR) offers us an environmentally benign and sustainable route. In this communication, we report that C-doped TiO_2_ nanoparticles act as an efficient electrocatalyst for the NRR with excellent selectivity. In 0.1 M Na_2_SO_4_, it achieves an NH_3_ yield of 16.22 μg h^−1^ mg_cat._^−1^ and a faradaic efficiency of 1.84% at −0.7 V *vs.* the reversible hydrogen electrode. Furthermore, this catalyst also shows good stability during electrolysis and recycling tests.

## Abstract 2

C-TiO_2_ is efficient for electrochemical N_2_ fixation to NH_3_ in 0.1 M Na_2_SO_4_, achieving a faradaic efficiency of 1.84% with an NH_3_ yield of 16.22 μg h^−1^ mg_cat._^−1^ at a potential of −0.7 V *vs* RHE.

## Graphical abstract

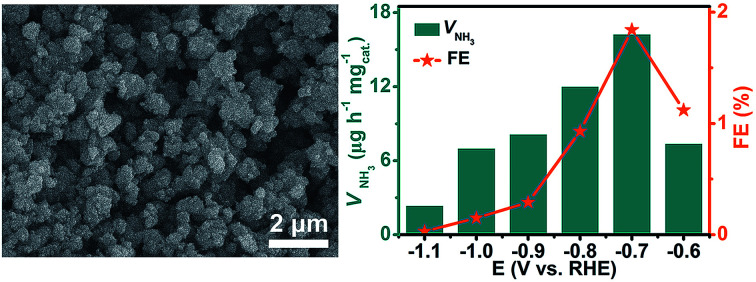

NH_3_ is an essential ingredient in the manufacture of fertilizers, medicaments, resins, dyes, explosives, *etc.*^1–4^ In 2017, total worldwide NH_3_ production exceeded 150 million tons, and the demand for NH_3_ continues to grow.^5^ Industrially, NH_3_ is produced almost *via* the Haber–Bosch process.^6^ In order to overcome the kinetic limitations of strong N≡N triple bonds, elevated temperature (350–550 °C) and high pressure (150–350 atm) are necessary throughout the whole process.^7–9^ Moreover, it not only consumes a large amount of energy, but inevitably leads to significant CO_2_ emission. So, it is imperative to develop an environmentally friendly process for the sustainable conversion of N_2_ to NH_3_.

Electrochemical NH_3_ synthesis from N_2_ and H_2_O is a promising candidate for artificial N_2_ fixation under ambient conditions due to its environment-friendly, convenient and low-cost characteristics.^10–15^ Although electrochemical reduction is feasible for achieving the conversion of N_2_ to NH_3_, it requires electrocatalysts for the N_2_ reduction reaction (NRR) to meet the challenge associated with N_2_ activation. Noble-metal catalysts such as Ru,^16^ Au,^17,18^ Ag,^19^ and Rh^20^ were reported as NRR catalysts with attractive catalytic performances, but the scarcity of these catalysts limits their wide application. Recently, transition metal oxides (TMOs)^21–26^ have attracted much attention as NRR electrocatalysts, as they are inexpensive and can be easily prepared on a large scale. Therefore, it is still highly desirable to develop TMOs for the NRR. TiO_2_ is nontoxic with a high thermal stability,^27^ but its low electronic conductivity hinders its electrocatalytic application.^28^ It has been reported that carbon doping can enhance the electronic conductivity of TiO_2_ and facilitate charge transfer from the bulk to the surface region,^29^ offering us a possible catalyst for the NRR, which, however, has not been explored before.

Herein, we report that C-doped TiO_2_ nanoparticles (C-TiO_2_) are effective for electrochemical N_2_ conversion to NH_3_ with excellent selectivity under ambient conditions. In 0.1 M Na_2_SO_4_, the catalyst achieves an NH_3_ yield of 16.22 μg h^−1^ mg_cat._^−1^ and a faradaic efficiency (FE) of 1.84% at −0.7 V *vs.* the reversible hydrogen electrode (RHE). Remarkably, it also demonstrates a high electrochemical stability. Compared with pristine TiO_2_ (NH_3_ yield: 8.49 μg h^−1^ mg_cat._^−1^; FE: 1.28%), C-TiO_2_ has a superior NRR performance. This result suggests that the introduction of carbon can enhance the electrocatalytic activity of TiO_2_.

C-TiO_2_ nanoparticles were prepared by a facile calcination assisted solvothermal method (see the ESI† for preparation details). Fig. 1a presents the X-ray diffraction (XRD) patterns of C-TiO_2_ and TiO_2_. The diffraction peaks at 25.3°, 37.8°, 48.0°, 53.9°, 55.1°, and 62.7° can be indexed to the (101), (004), (200), (105), (211), and (204) planes of anatase TiO_2_ (JCPDS no. 21-1272), respectively, which is similar to the pattern of C-TiO_2_. Thermal gravimetric analysis (Fig. S1†) demonstrated that the content of C was 2.97 wt%. Scanning electron microscopy (SEM) images (Fig. S2†) indicate that the crystallite size of C-TiO_2_ is smaller than that of TiO_2_. Fig. 1b shows a transmission electron microscopy (TEM) image which evidences the nanoparticle nature of C-TiO_2_. A high-resolution TEM (HRTEM) image (Fig. 1c) reveals a well-resolved lattice fringe with an interplanar distance of 0.35 nm, indexed to the (101) plane of C-TiO_2_. The selected area electron diffraction (SAED) pattern of C-TiO_2_ (Fig. 1d) exhibited four diffraction rings indexed to the (101), (004), (200) and (211) planes of the TiO_2_ phase.

**Fig. 1:**
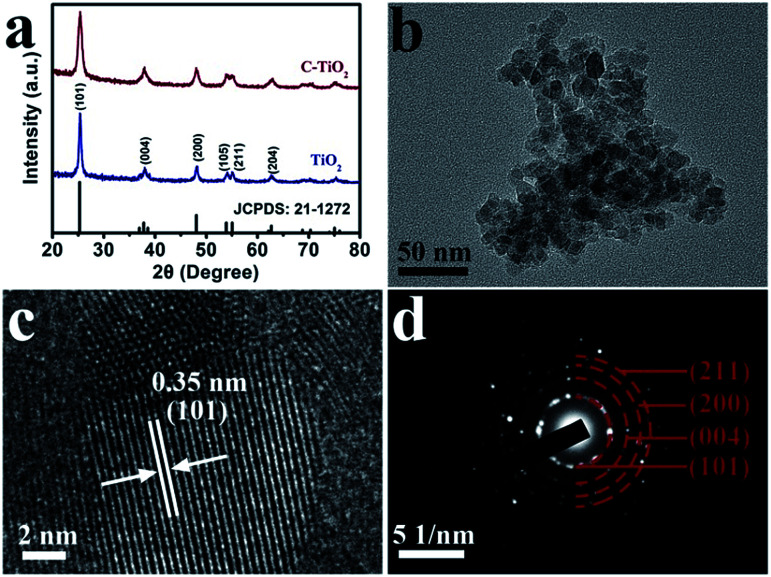
(a) XRD patterns for C-TiO_2_ and TiO_2_. (b) TEM and (c) HRTEM images for the C-TiO_2_ nanoparticles. (d) SAED pattern for C-TiO_2_.

Fig. 2a shows the X-ray photoelectron spectroscopy (XPS) survey spectrum of C-TiO_2_, which confirms the presence of Ti, C, and O elements. Fig. 2b presents the Ti 2p spectra for the C-TiO_2_ and TiO_2_ samples. The binding energies (BEs) of Ti 2p_3/2_ and Ti 2p_1/2_ for TiO_2_ are 458.38 and 464.07 eV, respectively.^30^ Compared to the TiO_2_ sample, the Ti 2p peaks of C-TiO_2_ show a positive shift of 0.3 eV, which could be attributed to lattice distortions.^31^Fig. 2c reveals the O 1s spectra for C-TiO_2_ which are in good agreement with those of pure TiO_2_. The BEs at 529.92 and 531.33 eV in the O 1s region are ascribed to the Ti–O–Ti (lattice oxygen) and O–H bonds in C–TiO_2_.^32,33^ For the C 1s XPS spectra (Fig. 2d), three peaks can be deconvoluted at around 284.76, 286.15, and 289.12 eV for C-TiO_2_. The peak at 284.76 eV could be attributed to the surface adventitious carbon.^30^ The two peaks at 286.15 and 289.12 eV are characteristic of the oxygen bound species C–O and Ti–O–C, respectively.^34^ This result indicates that carbon atoms substitute for some of the lattice titanium atoms and form a Ti–O–C structure.^30^ Compared with C-TiO_2_, only one C 1s XPS spectrum corresponding to C–C is observed for the TiO_2_ sample, further confirming the existence of C in C-TiO_2_. In addition, the ultraviolet-visible (UV-vis) absorption spectra and the corresponding Kubelka–Munk plots of C-TiO_2_ and TiO_2_ are displayed in Fig. S3.† The band gap energies of C-TiO_2_ (2.79 eV) and TiO_2_ (2.96 eV) were determined by the intercept of the plots of (*αhν*)^1/2^*versus* photon energy (*hν*),^35^ indicating a narrower band gap after C doping. The enhancement of visible light absorption for C-TiO_2_ and TiO_2_ should be attributed to the carbon doping in the TiO_2_ lattice, which would introduce a series of localized occupied states into the band gap of the TiO_2_ lattice, leading to a strong visible light absorption.^36^ All of the above results strongly support the successful preparation of C-TiO_2_ nanoparticles.

**Fig. 2:**
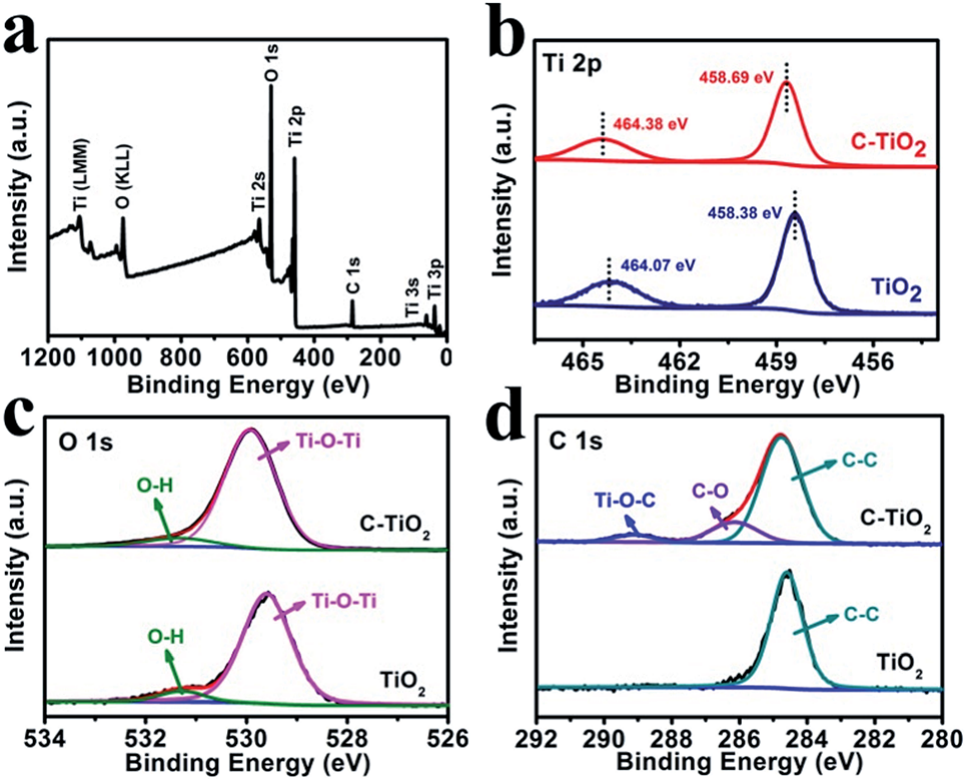
(a) XPS survey spectrum for C-TiO_2_. XPS spectra of C-TiO_2_ and TiO_2_ in the (b) Ti 2p, (c) O 1s and (d) C 1s regions.

The electrocatalytic NRR performance of C-TiO_2_ was tested using a typical two-compartment and three-electrode device as the reaction vessel. C-TiO_2_ was deposited on carbon paper (C-TiO_2_/CP with a C-TiO_2_ loading of 0.10 mg) for the test. All of the potentials for the NRR were reported on the RHE scale. The produced NH_3_ was detected by spectrophotometry with salicylic acid.^37^ The relevant calibration curves are shown in Fig. S4.† The chronoamperometry curves at the corresponding potentials in N_2_-saturated 0.1 M Na_2_SO_4_ are displayed in Fig. 3a, which can directly express the relationship between current density and time during the whole test process. Fig. 3b presents the UV-vis absorption spectra of the electrolyte stained with indophenol indicator after 2 h electrolysis at a series of potentials, and the values of absorbance at 660 nm were used to calculate the concentrations of the generated NH_3_ at different applied potentials according to the calibration curve of NH_3_. Combined with the collected data, the final results including the NH_3_ yields and FEs under various potentials were calculated and are plotted in Fig. 3c. Both the NH_3_ yields and FEs increase as the negative potential rises to −0.7 V, which is the optimum potential point when the NH_3_ yield and FE are 16.22 μg h^−1^ mg_cat._^−1^ and 1.84%, respectively. After that, as the potential continually increases, both the NH_3_ yields and FEs decrease significantly which is mainly caused by the competitive hydrogen evolution reaction. For comparison, the pure TiO_2_ sample was tested under the same conditions and the corresponding results are presented in Fig. 3d. It is worth noting that the performance of C-TiO_2_ is evidently better than that of pure TiO_2_. The superior NRR performance of C-TiO_2_ can be rationally attributed to the C-TiO_2_ nanoparticles having more exposed active sites (Fig. S5†), enabling more effective utilization of them as electrocatalysts. The enhanced conductivity of C-TiO_2_ also contributes to its higher catalytic activity. The charge transfer resistance related to the electrocatalytic kinetics can be determined from the diameter of the semicircles in the low frequency zone.^38^ Electrochemical impedance spectroscopy data (Fig. S6†) show that C-TiO_2_/CP possesses a smaller radius of the semicircle compared to TiO_2_/CP, suggesting that the C-TiO_2_ sample has a lower charge transfer resistance^39^ and thus faster NRR kinetics. Meanwhile, C-TiO_2_ shows a higher performance than some of the previously reported NRR electrocatalysts.^40–44^ More detailed comparisons are listed in Table S1.†

**Fig. 3:**
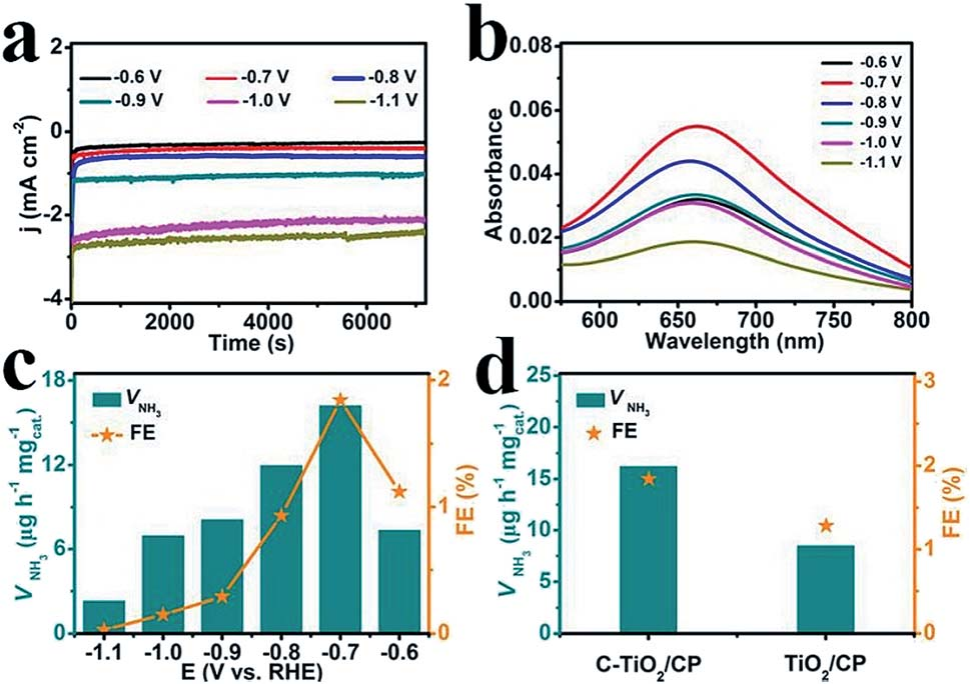
(a) Chronoamperometry curves at the corresponding potentials in N_2_-saturated 0.1 M Na_2_SO_4_. (b) UV-vis absorption spectra of the electrolytes stained with indophenol indicator after 2 h electrolysis at a series of potentials. (c) The NH_3_ yields and FEs of C-TiO_2_ for the NRR at a series of potentials. (d) The amount of NH_3_ with different electrodes at −0.7 V after 2 h electrolysis under ambient conditions.

To prove that NH_3_ was generated *via* the N_2_ reduction process of C-TiO_2_, three sets of control experiments were carried out: (1) immersing the samples in Ar-saturated solution at −0.7 V for 2 h; (2) immersing the samples in N_2_-saturated solution at an open circuit potential for 2 h; and (3) immersing the samples at −0.7 V with alternating 2 h cycles between N_2_-saturated and Ar-saturated solutions, for a total of 12 h. As shown in Fig. 4a and Fig. S7,† a trace amount of NH_3_ production was detected under Ar-saturated solution and an open circuit potential. Combined with Fig. S8,† this result indicates that only N_2_ provides the nitrogen source to NH_3_. Moreover, controlled trials were carried out to investigate the performance of bare CP. The relevant UV-vis absorption spectra are displayed in Fig. S9.† The results show the poor electrocatalytic activity of bare CP, indicating that C-TiO_2_ is an active material for the NRR (Fig. 4b). In addition, stable performance is another important indicator for evaluating catalysts. Recycling tests were performed in N_2_-saturated 0.1 M Na_2_SO_4_ 6 times and the results are shown in Fig. 4c. The NH_3_ yield and FE results show no obvious fluctuation over the whole process, suggesting that C-TiO_2_ possesses a stable NRR performance. Moreover, only a slight fluctuation of current density is observed at −0.7 V after 24 h electrolysis, further suggesting an excellent electrochemical stability.

**Fig. 4:**
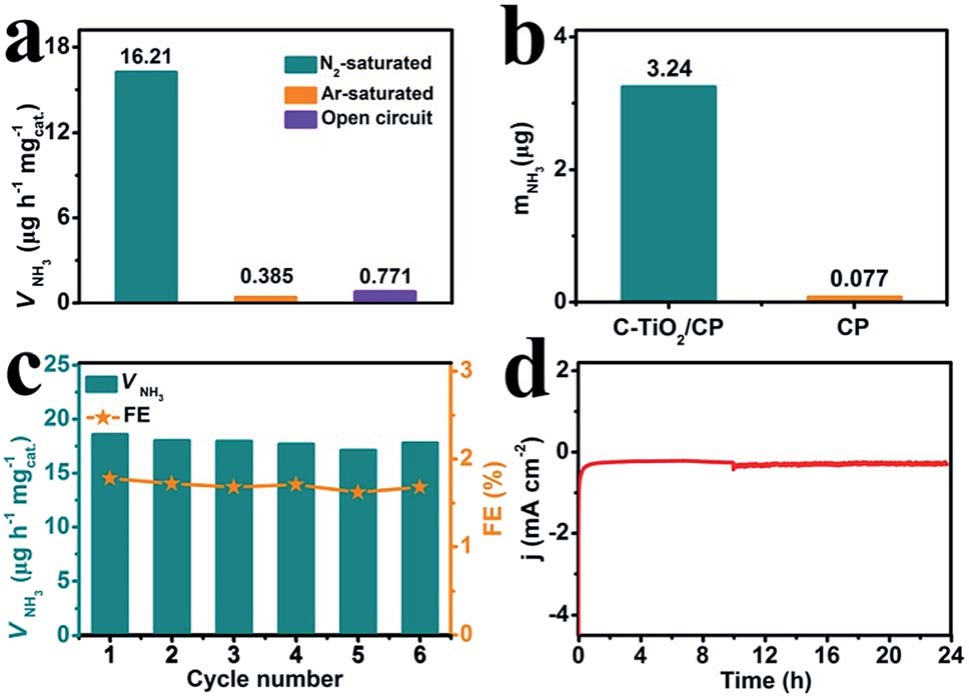
(a) NH_3_ yields for C-TiO_2_ under different conditions. (b) The amount of NH_3_ with different electrodes at −0.7 V after 2 h electrolysis under ambient conditions. (c) NH_3_ yields and FEs at a potential of −0.7 V during 6 recycling tests. (d) Chronoamperometry curve at a potential of −0.7 V using a C-TiO_2_/CP catalyst for 24 h.

Hydrazine (N_2_H_4_), as a possible by-product in the NRR test, was detected by the method of Watt and Chrisp.^45^ The relevant calibration curves are displayed in Fig. S10.† The UV-vis absorption spectra of N_2_H_4_ after 2 h electrolysis in a N_2_ atmosphere at a series of potentials are shown in Fig. S11.† The concentrations of the possible by-product N_2_H_4_ are determined according to the values of absorbance at 455 nm. The results demonstrated that no N_2_H_4_ was detected at all potentials, implying the excellent selectivity of C-TiO_2_ as an NRR electrocatalyst.

In summary, C-TiO_2_ nanoparticles have been proven as an effective non-noble-metal electrocatalyst for the NRR at moderate temperatures and atmospheric pressure. This electrocatalyst achieves an NH_3_ yield of 16.22 μg h^−1^ mg_cat._^−1^ and a FE of 1.84% at −0.7 V *vs.* RHE in 0.1 M Na_2_SO_4_. It also exhibits excellent selectivity and satisfactory electrochemical stability during the process of electrochemical NH_3_ synthesis under ambient conditions. This work not only offers us an attractive earth-abundant electrocatalyst for the NRR, but also opens up an exciting new avenue for the design and development of doped Ti-based catalysts^46,47^ with enhanced performances toward electrocatalytic N_2_ and nitrite^48^ reduction for applications.

## Conflicts of interest

There are no conflicts to declare.

## Supplementary Material

NA-001-C8NA00300A-s001
