# Supplementary material for: Enabling the electrocatalytic fixation of N2 to NH3 by C-doped TiO2 nanoparticles under ambient conditions
Source: Nanoscale Adv. 2018 Nov 21;1(3):961–4. doi: 10.1039/c8na00300a (PMC9473171; doi:10.1039/c8na00300a)
Supplement: NA-001-C8NA00300A-s001 [file NA-001-C8NA00300A-s001.pdf]

Electronic Supplementary Material (ESI) for Nanoscale Advances.
This journal is © The Royal Society of Chemistry 2018

Electronic Supplementary Information

Experimental Section

Materials: Titanium butoxide ($C_{16}H_{36}O_4Ti$), Para-(dimethylamino) benzaldehyde ($C_9H_{11}NO$), sodium nitroferricyanide (III) dihydrate ($Na_2Fe(CN)_5NO \cdot 2H_2O$), hydrogen peroxide ($H_2O_2$) and Nafion (5 wt%) were purchased from Aladdin Ltd. (Shanghai, China). Sulfuric acid (H_2SO_4), ammonium chloride (NH_4Cl), hydrazine hydrate ($N_2H_4 \cdot H_2O$), sodium hypochlorite (NaClO), sodium hydroxide (NaOH), sodium salicylate ($C_7H_5O_3Na$), sodium sulfate (Na_2SO_4), hydrochloric acid (HCl), ethanol (CH_3CH_2OH) and carbon paper were bought from Beijing Chemical Corporation. The water used throughout all experiments was purified through a Millipore system.

Preparation of C-TiO_2 nanoparticles: Carbon doped TiO_2 nanoparticles have been prepared by a facile calcination assisted hydrothermal method. In a typical hydrothermal synthesis process, 13mL titanium butoxide was added dropwise to 35mL water/ethanol (5:30) mixed solution with continuous stirring for 30 min to form a milk-white solution. Then, the solution was transferred into a 50 mL Teflon-lined stainless steel autoclave and heated at 190℃ for 2h. Subsequently, the products were centrifuged, washed with ultrapure water and ethanol three times, respectively, followed by drying in a vacuum oven at 60℃ overnight. The as-prepared white precursor powder was calcined at 265℃ for 1 h in a muffle furnace to obtain the final products. For comparison, the pure TiO_2 sample was prepared under the same condition without ethanol and calcination.

Preparation of C-TiO_2/CP electrode: 10 mg C-TiO_2 powders and 20 μL of Nafion solution (5 wt%) were dispersed in 980 μL mixed solution contain 735 μL ethanol and 245 μL H_2O by 2 h sonication to form a homogeneous ink. Then 10 μL catalyst ink was loaded on a 1 × 1 cm^2 carbon paper and dried under ambient condition.

Characterizations: XRD data were recorded using a Shimazu XRD-6100

diffractometer with Cu Kα radiation (40 kV, 30 mA) of wavelength 0.154 nm (SHIMADZU, Japan). SEM images were obtained from a tungsten lamp-equipped SU3500 scanning electron microscope at an accelerating voltage of 20 kV (HITACHI, Japan). TEM images were collected from a HITACHI H-8100 electron microscopy (Hitachi, Tokyo, Japan) operated at 200kV. XPS data were acquired on an ESCALABMK II X-ray photoelectron spectrometer using Mg as the exciting source. The data of absorbance and UV-vis diffuse reflectance spectra were measured on a SHIMADZU UV-1800 ultraviolet-visible spectrophotometer. Thermogravimetric analysis (TGA) was performed on a Perkin-Elmer Model Pyris1 TGA apparatus at a heating rate of 10 °C min^{-1} in nitrogen atmosphere.

Electrocatalytic N_2 reduction measurements: The N_2 reduction experiments were carried out in a two-compartment cell under ambient condition, which was separated by Nafion 211 membrane. The membrane was treated in H_2O_2 (5%) aqueous solution at 80 °C for 1 h and dipped in 0.1 M H_2SO_4 at 80°C for another 1 h. And finally, the membrane was treated in ultrapure water at 80°C for 6h. The electrochemical experiments were carried out with a CHI 660E electrochemical analyzer using a three-electrode configuration with C-TiO_2/CP electrode, graphite rod and Ag/AgCl electrode (saturated KCl electrolyte) as working electrode, counter electrode and reference electrode, respectively. The potentials reported in this work were converted to reversible hydrogen electrode (RHE) scale via calibration with the following equation: in 0.1 M Na_2SO_4 aqueous solution, E (vs. RHE) = E (vs. Ag/AgCl) + 0.059 × pH + 0.197 V. The presented current density was normalized to the geometric surface area. For electrochemical N_2 reduction, chronoamperometry tests were conducted in N_2-saturated 0.1 M Na_2SO_4 solution(40mL). All experiments were operated under room temperature.

Determination of NH_3: Concentration of produced NH_3 was spectrophotometrically determined by spectrophotometry measurement with salicylic acid.[1] Typically, 4 mL of the electrolyte was taken from the cathodic chamber. Then 50 uL of NaClO (4.5%) and NaOH (0.75 M), 500 μL of $C_7H_5O_3Na$ (0.4 M) and NaOH (0.32 M) and 50 uL of

1% $Na_2Fe(CN)_5NO \cdot 2H_2O$ were successively added into the above solution. Absorbance measurements were performed after 2 h at a wavelength of 660 nm. The concentration-absorbance curves were calibrated using standard NH_4^+ solution with a serious of concentrations. The fitting curve (y = 0.519x + 0.012, R^2 = 0.999) shows good linear relation of absorbance value with NH_4Cl concentration.

Determination of N_2H_4: The N_2H_4 presented in the electrolyte was estimated by the method of Watt and Chrisp.[2] A mixed solution of $C_9H_{11}NO$ (5.99 g), HCl (concentrated, 30 mL) and ethanol (300 mL) was used as a color reagent. Typically, 5 mL electrolyte was removed from the cathodic chamber, after that, added into 5 mL above prepared color reagent and stirring 20 min at room temperature. The absorbance of the resulting solution was measured at 455 nm. The concentration absorbance curves were calibrated using standard $N_2H_4 \cdot H_2O$ solution with a series of concentrations. The fitting curve (y = 0.566x + 0.037, R^2 = 0.999) shows good linear relation of absorbance value with N_2H_4 concentration.

Determination of FE: The Faradaic efficiency (FE) for N_2 reduction was defined as the amount of electric charge used for synthesizing NH_3 divided the total charge passed through the electrodes during the electrolysis. The total amount of NH_3 produced was measured using colorimetric methods. Assuming three electrons were needed to produce one NH_3 molecule, the FE could be calculated as follows:

$$FE = \frac{3F \times [NH_4^+] \times V}{17 \times Q}$$

The rate of NH_3 formation was calculated using the following equation:

$$V_{NH_3} = \frac{[NH_4^+] \times V}{t \times m_{cat.}}$$

Where F is the Faraday constant, $[NH_4^+]$ is the measured NH_4^+ concentration, V is the volume of the electrolyte in the cathodic chamber, Q is the total quantity of applied electricity; t is the reduction time; $m_{cat.}$ is the loaded mass of catalyst on carbon paper.

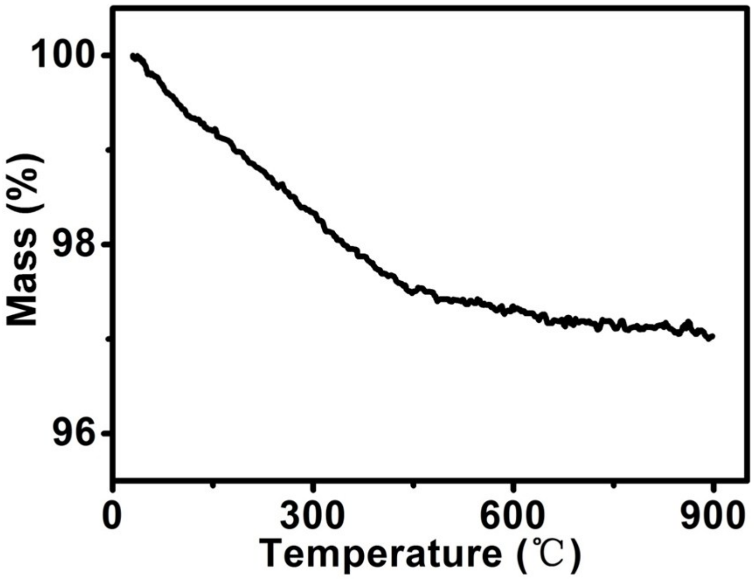

Fig. S1. TGA curve of C-TiO_2.

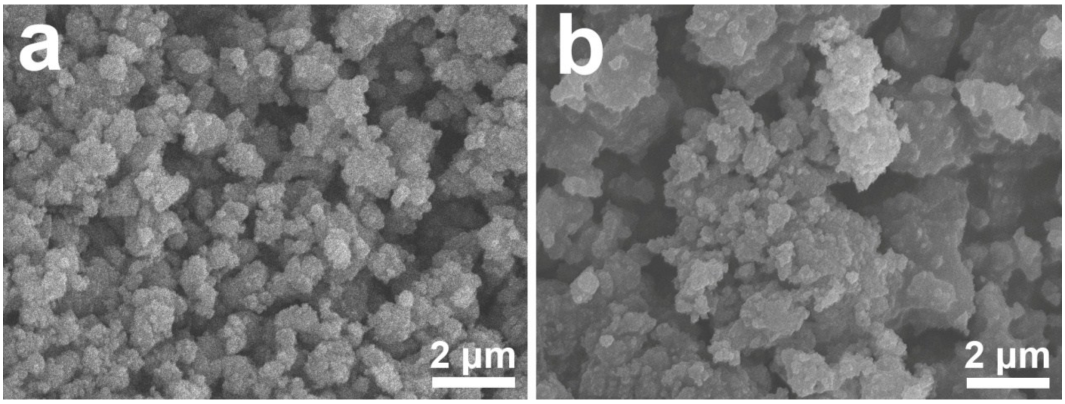

Fig. S2. SEM images of C-TiO_2 and TiO_2.

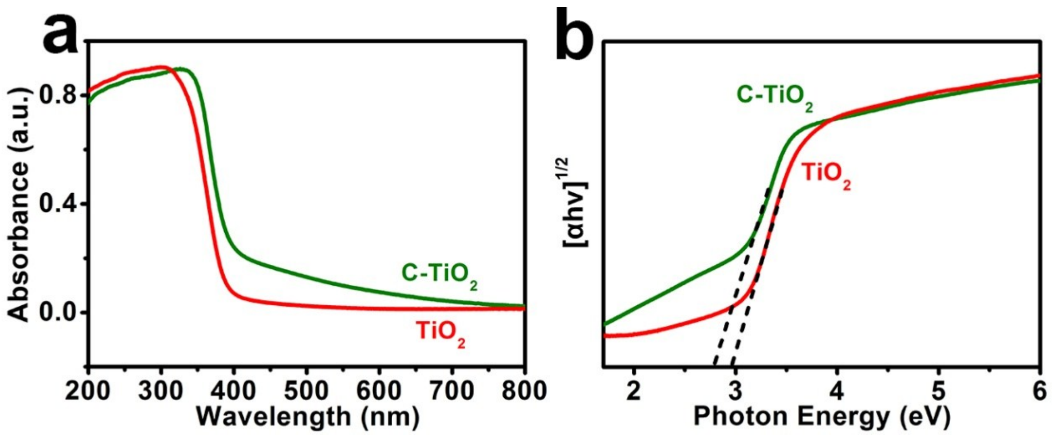

Fig. S3. (a) UV–vis absorption spectra of C-TiO_2 and TiO_2. (b) the Kubelka–Munk plots for the corresponding reflectance spectra of samples.

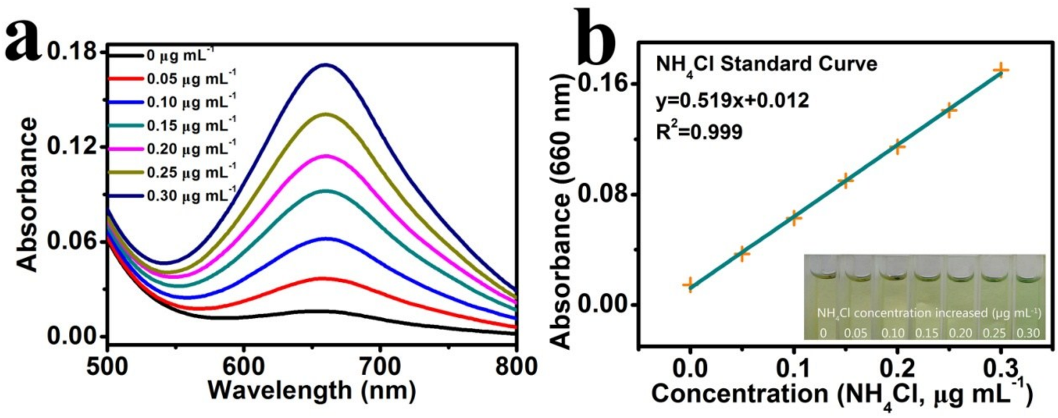

Fig. S4. (a) UV–vis absorption spectra of various NH_4^+ concentrations after incubated for 2 hours at room temperature. (b) Calibration curve used for calculation of NH_4^+ concentrations.

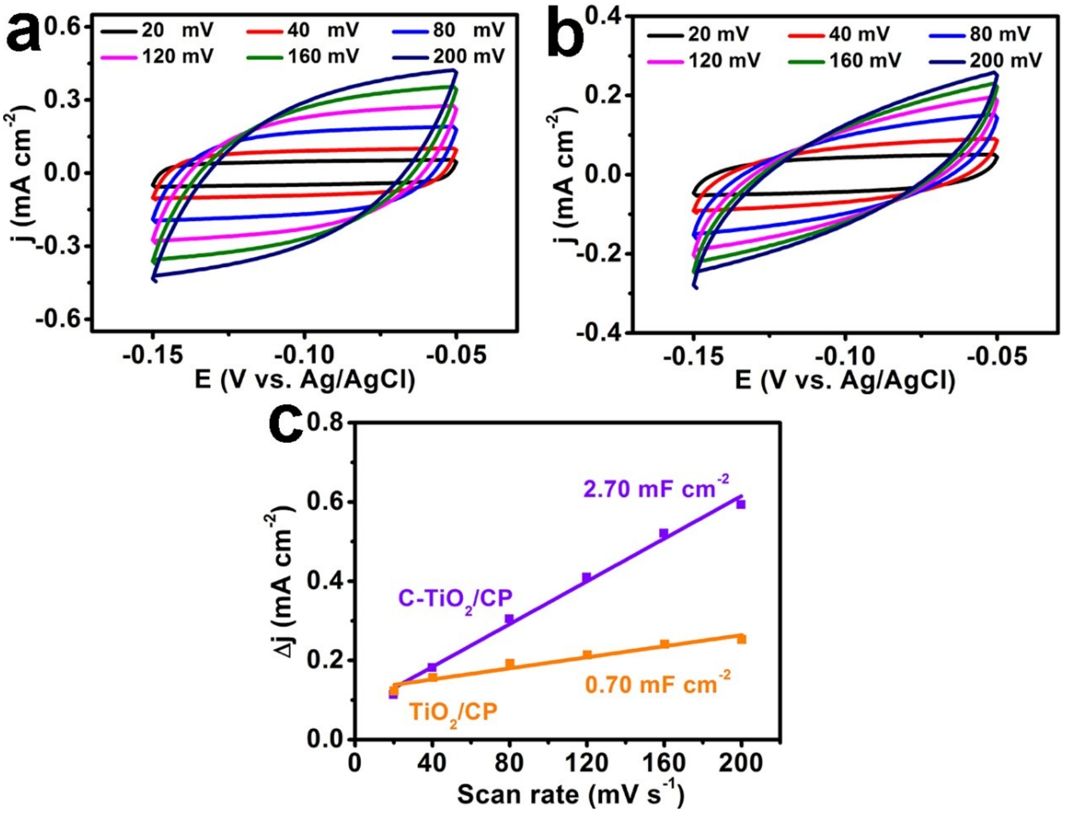

Fig. S5. CVs of (a) C-TiO_2/CP and (b) TiO_2/CP with various scan rates (20-200 mV s^{-1}) in the region of –0.05 to –0.15 V vs. Ag/AgCl. (c) The capacitive current densities at –0.10 V vs. Ag/AgCl as a function of scan rates for C-TiO_2/CP and TiO_2/CP.

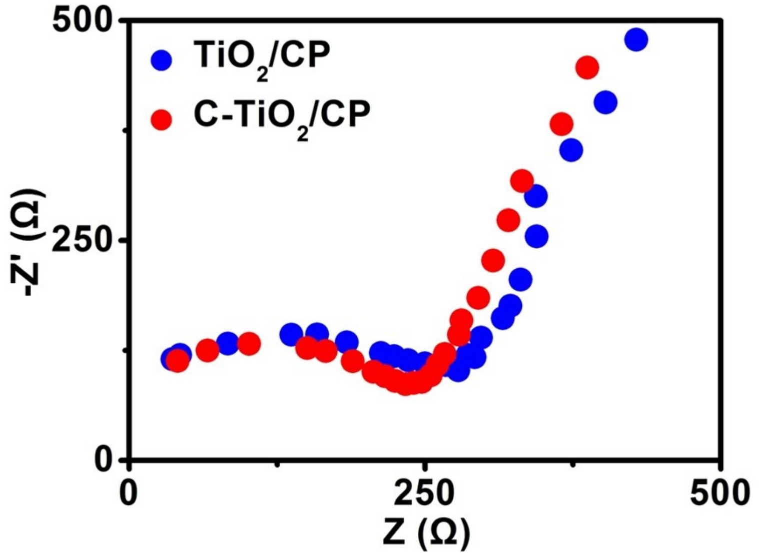

Fig. S6. Nyquist plots of C-TiO_2/CP and TiO_2/CP in the frequency range from 1000 kHz to 1Hz with a voltage amplitude of 5 mV, and all the three electrodes are in one compartment cell being full of 0.1 M Na_2SO_4 solution.

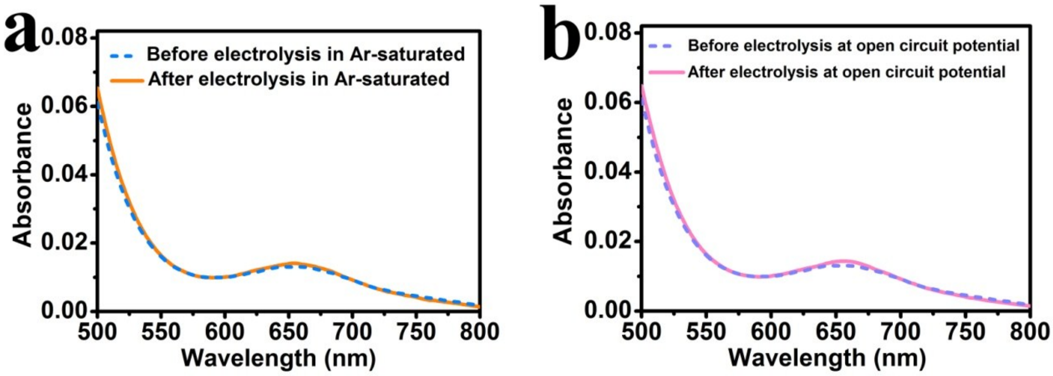

Fig. S7. UV–vis absorption spectra of the electrolytes stained with indicator before and after 2 h electrolysis (a) at the potential of –0.7 V in Ar-saturated solution (b) at open circuit potential in N_2-saturated solution on C-TiO_2/CP.

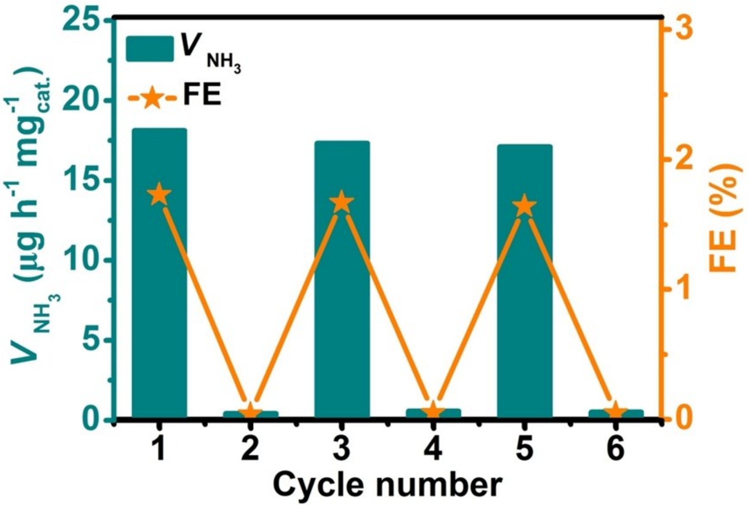

Fig. S8. NH_3 yields and FEs of C-TiO_2 at the potential of –0.7 V with alternating 2 h cycles between N_2-saturated and Ar-saturated solutions for NRR.

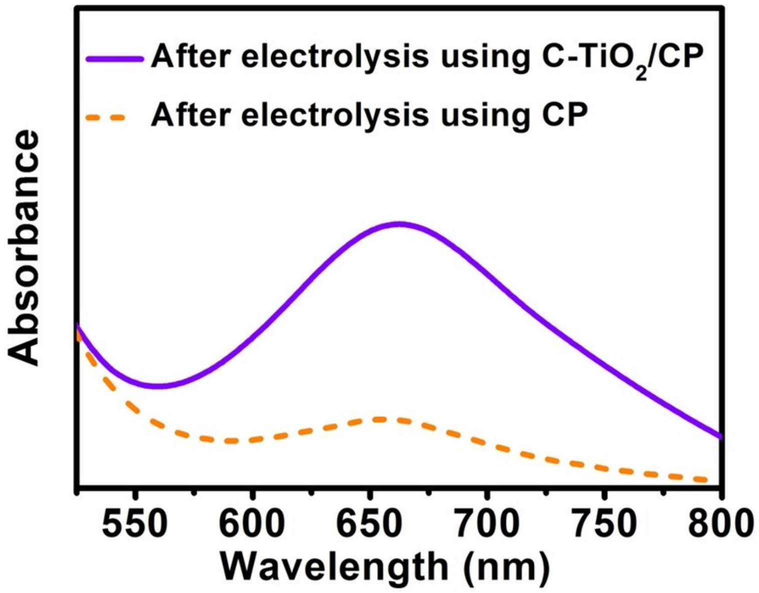

Fig. S9. UV–vis absorption spectra of the electrolytes estimated by the method of Watt and Chrisp before and after 2 h electrolysis in N_2-saturated solution at –0.70 V.

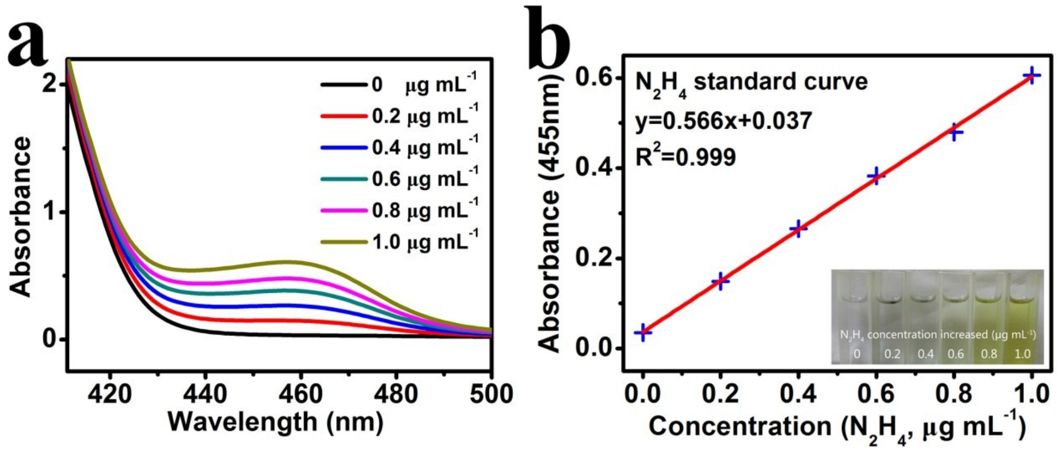

Fig. S10. (a) UV–vis absorption spectra of various N_2H_4 concentrations after incubated for 20 min at room temperature. (b) Calibration curve used for calculation of N_2H_4 concentrations.

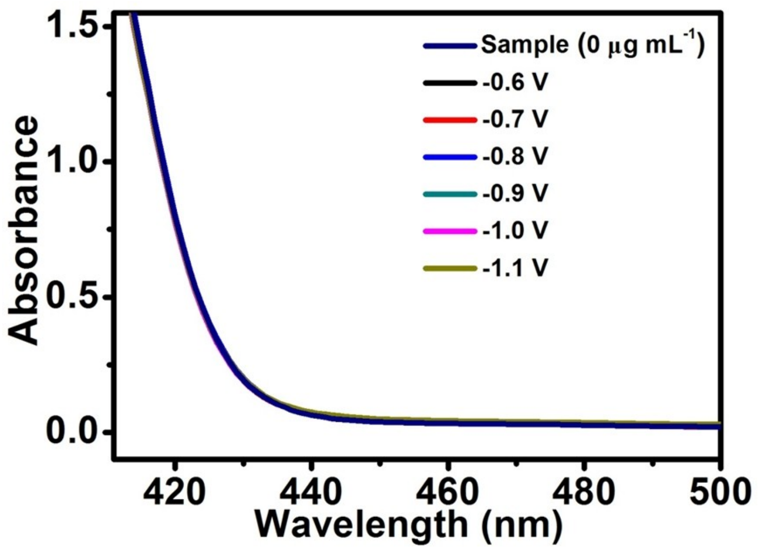

Fig. S11. UV–vis spectra of the electrolyte estimated by the method of Watt and Chrisp before and after 2 h electrolysis in N_2 atmosphere at a series of potentials under ambient conditions.

Table S1. Comparison of ambient N_2 reduction performance for C-TiO_2 nanoparticles with other aqueous-based NRR electrocatalysts.

Catalyst	Electrolyte	NH_3 yield	FE (%)	Ref.
C-TiO_2	0.1 M Na_2SO_4	16.22 μg h^{-1} $mg^{-1}_{cat.}$	1.84	This work
N-doped porous carbon	0.05 M H_2SO_4	23.8 μg h^{-1} $mg^{-1}_{cat.}$	1.42	3
Mo nanofilm	0.01 M H_2SO_4	1.89 μg h^{-1} cm^{-2}	0.72	4
γ-Fe_2O_3	0.1 M KOH	0.212 μg h^{-1} $mg^{-1}_{cat.}$	1.9	5
$Pd_{0.2}Cu_{0.8}$/rGO	0.1 M KOH	2.80 μg h^{-1} $mg^{-1}_{cat.}$	4.5	6
Fe_2O_3 nanorods	0.1 M Na_2SO_4	15.9 μg h^{-1} $mg^{-1}_{cat.}$	0.94	7
PEBCD/C	0.5 M Li_2SO_4	1.58 μg h^{-1} cm^{-2}	2.85	8
Fe_2O_3-CNT	$KHCO_3$	0.22 μg h^{-1} cm^{-2}	0.15	9
Au nanorods	0.1 M KOH	6.042 μg h^{-1} $mg^{-1}_{cat.}$	4.0	10
TA-reduced Au/TiO_2	0.1 M HCl	21.4 μg h^{-1} $mg^{-1}_{cat.}$	8.11	11
α-Au/CeO_x-RGO	0.1 M HCl	8.31 μg h^{-1} $mg^{-1}_{cat.}$	10.1	12
MoN	0.1 M HCl	3.01×10^{-10} mo1 s^{-1} cm^{-2}	1.15	13
Ru/C	2.0 M KOH	0.21 μg h^{-1} cm^{-2}	0.28	14
MoS_2/CC	0.1 M Na_2SO_4	0.02 μg h^{-1} cm^{-2}	1.17	15
Fe_3O_4/Ti	0.1 M Na_2SO_4	0.012 μg h^{-1} cm^{-2}	2.6	16
$Bi_4V_2O_{11}$/CeO_2	0.1 M HCl	23.21 μg h^{-1} $mg^{-1}_{cat.}$	10.16	17
MoO_3	0.1 M HCl	29.43 μg h^{-1} $mg^{-1}_{cat.}$	1.9	18
VN/TM	0.1 M HCl	5.14 μg h^{-1} cm^{-2}	2.25	19
Nb_2O_5 nanofiber	0.1 M HCl	43.6 μg h^{-1} $mg^{-1}_{cat.}$	9.26	20
TiO_2	0.1 M Na_2SO_4	9.16×10^{-11} mol s^{-1}·cm^{-2}	2.5	21
TiO_2-rGO	0.1 M Na_2SO_4	15.13 μg h^{-1} $mg^{-1}_{cat.}$	3.3	22

References

1 D. Zhu, L. Zhang, R. E. Ruther and R. J. Hamers, *Nat. Mater.*, 2013, **12**, 836–841.

2 G. W. Watt and J. D. Chrisp, *Anal. Chem.*, 1952, **24**, 2006–2008.

3 Y. Liu, Y. Su, X. Quan, X. Fan, S. Chen, H. Yu, H. Zhao, Y. Zhang and J. Zhao, *ACS Catal.*, 2018, **8**, 1186−1191.

4 D. Yang, T. Chen and Z. Wang, *J. Mater. Chem. A*, 2017, **5**, 18967–18971.

5 J. Kong, A. Lim, C. Yoon, J. H. Jang, H. C. Ham, J. Han, S. Nam, D. Kim, Y. Sung, J. Choi and H. S. Park, *ACS Sustainable Chem. Eng.*, 2017, **5**, 10986–10995.

6 M. Shi, D. Bao, S. Li, B. Wulan, J. Yan and Q. Jiang, *Adv. Energy Mater.*, 2018, **8**, 1800124.

7 X. Xiang, Z. Wang, X. Shi, M. Fan and X. Sun, *ChemCatChem.*, 2018, **10**, 1–7.

8 G. Chen, X. Cao, S. Wu, X. Zeng, L. Ding, M. Zhu and H. Wang, *J. Am. Chem. Soc.*, 2017, **139**, 9771–9774.

9 S. Chen, S. Perathoner, C. Ampelli, C. Mebrahtu, D. Su and G. Centi, *Angew. Chem., Int. Ed.*, 2017, **56**, 2699–2703.

10 D. Bao, Q. Zhang, F. Meng, H. Zhong, M. Shi, Y. Zhang, J. Yan, Q. Jiang and X. Zhang, *Adv. Mater.*, 2017, **29**, 1604799.

11 M. Shi, D. Bao, B. Wulan, Y. Li, Y. Zhang, J. Yan and Q. Jiang, *Adv. Mater.*, 2017, **29**, 1606550.

12 S. Li, D. Bao, M. Shi, B. Wulan, J. Yan and Q. Jiang, *Adv. Mater.*, 2017, **29**, 1700001.

13 L. Zhang, X. Ji, X. Ren, Y. Luo, X. Shi, A. M. Asiri, B. Zheng and X. Sun, *ACS Sustainable Chem. Eng.*, 2018, **6**, 9550–9554.

14 V. Kordali, G. Kyriacou and C. Lambrou, *Chem. Commun.*, 2000, **17**, 1673–1674.

15 L. Zhang, X. Ji, X. Ren, Y. Ma, X. Shi, Z. Tian, A. M. Asiri, L. Chen, B. Tang

and X. Sun, *Adv. Mater.*, 2018, **30**, 1800191.

16 Q. Liu, X. Zhang, B. Zhang, Y. Luo, G. Cui, F. Xie and X. Sun, *Nanoscale*, 2018, **10**, 14386–14389.

17 C. Lv, C. Yan, G. Chen, Y. Ding, J. Sun, Y. Zhou and G. Yu, *Angew. Chem.*, *Int. Ed.*, 2018, **57**, 6073–6076.

18 J. Han, X. Ji, X. Ren, G. Cui, L. Li, F. Xie, H. Wang, B. Li and X. Sun, *J. Mater. Chem. A*, 2018, **6**, 12974–12977.

19 R. Zhang, Y. Zhang, X. Ren, G. Cui, A. M. Asiri, B. Zheng and X. Sun, *ACS Sustainable Chem. Eng.*, 2018, **6**, 9545–9549.

20 J. Han, Z. Liu, Y. Ma, G. Cui, F. Xie, F. Wang, Y. Wu, S. Gao, Y. Xu and X. Sun, *Nano Energy*, 2018, **52**, 264–270.

21 R. Zhang, X. Ren, X. Shi, F. Xie, B. Zheng, X. Guo and X. Sun, *ACS Appl. Mater. Interfaces*, 2018, ***10***, 28251–28255.

22 X. Zhang, Q. Liu, X. Shi, A. M. Asiri, Y. Luo, X. Sun and T. Li, *J. Mater. Chem. A*, 2018, **6**, 17303–17306.
